# The pill you don’t have to take that is still effective: neural correlates of imaginary placebo intake for regulating disgust

**DOI:** 10.1093/scan/nsae021

**Published:** 2024-03-07

**Authors:** Anne Schienle, Wolfgang Kogler, Arved Seibel, Albert Wabnegger

**Affiliations:** Department of Clinical Psychology, University of Graz, Universitaetsplatz 2, Graz 8010, Austria; Department of Clinical Psychology, University of Graz, Universitaetsplatz 2, Graz 8010, Austria; Department of Clinical Psychology, University of Graz, Universitaetsplatz 2, Graz 8010, Austria; Department of Clinical Psychology, University of Graz, Universitaetsplatz 2, Graz 8010, Austria

**Keywords:** open-label placebo, imagery, disgust, plausibility, fMRI

## Abstract

A commonly established protocol for the administration of open-label placebos (OLPs)—placebos honestly prescribed—emphasizes the necessity of ingesting the pill for the placebo effect to manifest. The current functional magnetic resonance imaging study used a novel approach to OLP administration: the imaginary intake of an OLP pill for regulating disgust. A total of 99 females were randomly allocated to one of three groups that either swallowed a placebo pill (OLP Pill), imagined the intake of a placebo pill (Imaginary Pill) or passively viewed (PV) repulsive and neutral images. The imaginary pill reduced reported disgust more effectively than the OLP pill and was also perceived as a more plausible method to reduce emotional distress. Relative to the OLP pill, the imaginary pill lowered neural activity in a region of interest involved in disgust processing: the pallidum. No significant differences in brain activation were found when comparing the OLP pill with PV. These findings highlight that imagining the intake of an OLP emerged as a superior method for regulating feelings of disgust compared to the actual ingestion of a placebo pill. The study’s innovative approach sheds new light on the potential of placebo interventions in emotion regulation.

## Introduction

In recent years, a new area of placebo research has emerged which examines the effects of so-called open-label placebos (OLPs). OLPs are administered without deception. The recipients are told that they are receiving an inert treatment, such as a pill not containing any active ingredients (Colloca and Howick, 2018). This OLP instruction is usually combined with the provision of further information about placebos and how they work. One such instruction, developed by Kaptchuk *et al*. (2010) as part of a clinical trial, has been used in numerous OLP studies. This instruction includes the following points: ‘(1) the placebo effect is powerful, (2) the body can automatically respond to taking placebo pills like Pavlov’s dogs who salivated when they heard a bell, (3) a positive attitude helps but is not necessary, and (4) taking the pills faithfully is critical.’ (p. 2).

One aspect of this rationale, the notion that taking the pill is a critical component of the OLP effect, has not yet been systemically investigated. This is surprising since this part of the instruction is counter-intuitive. The placebo recipients are asked to swallow an ‘empty’ capsule. This is like asking a person to drink from an empty glass (as one of our study participants once phrased it). To the best of our knowledge, there has been only one OLP study up until now that has systematically varied OLP intake; this was carried out through the inclusion of two conditions, one with and one without the actual ingestion of a placebo pill (Buergler *et al*., 2023a). In that study, healthy students with self-reported test anxiety were randomized either to a 3-week intervention with a daily intake of an OLP pill, an imaginary pill or a control condition (without a pill). Both placebo interventions were similarly efficacious in lowering test anxiety.

Based on this finding, the present functional magnetic resonance imaging (fMRI) study investigated the neural correlates of imaginary OLP treatment. Participants were randomly allocated to one of three groups that were presented with disgusting and neutral images. Two groups, with subsequent real *vs* imagined pill intake, first received information on placebos and how they work (with an emphasis on research findings from neuroscience). A third group (control group with passive picture viewing; PV) was provided with research findings from affective neuroscience but received no placebo information. The OLP groups were both instructed that the placebo can reduce disgust feelings. Whereas the ‘OLP Pill’ group was asked to swallow the placebo pill, the ‘Imaginary Pill’ group was told that the placebo does not have to be orally taken to be effective.

The exploratory research question of the current fMRI investigation focused on possible group differences (OLP Pill, Imaginary Pill, and PV) in brain activation during the viewing of disgusting pictures. To the best of our knowledge, no fMRI investigation has compared the effects of different OLP interventions before. Two previous fMRI studies (with healthy participants) found that reduced emotional distress after administering an OLP (with actual intake) was accompanied by activation changes in prefrontal cortex regions concerned with emotion regulation (e.g. ventrolateral prefrontal cortex, anterior cingulate cortex; Schaefer *et al*., 2023; Schienle *et al*., 2023). Moreover, in a study on disgust regulation, the OLP reduced activation in the insula and pallidum. These regions play an important role in decoding disgust signals from different modalities (Schienle *et al*., 2023).

Very similarly, fMRI studies that have used deceptive placebos for reducing emotional distress in healthy participants have identified frontal brain regions involved in cognitive control (e.g. ventrolateral/ dorsolateral prefrontal cortex [VLPFC, DLPFC)], as well as the insula and amygdala, as mediators of the placebo effect (Petrovic *et al*., 2005; Schienle *et al*., 2014, 2017; Meyer *et al*., 2015, 2019; Koban *et al*., 2017).

In the current study, we focused on differences in brain activity between participants with real *vs* imagined OLP intake. The selected regions of interest (VLPFC/DLPFC, insula, amygdala, pallidum) were based on the previous findings of fMRI studies on visual disgust processing and disgust regulation in healthy participants (Wicker *et al*., 2003; Khan *et al*., 2020; Schienle *et al*., 2023).

## Materials and methods

### Participants

A total of 99 females (mean age = 23.2 years, *SD* = 6.15) participated in this study. They were recruited via announcements at the university campus and through social media. Inclusion criteria were a minimum age of 18 years and female sex. We investigated a female sample because of gender differences in placebo reactivity (Enck and Klosterhalfen, 2019) and disgust propensity (the temporally stable tendency of a person to experience disgust across different situations (Schienle *et al*., 2020). Exclusion criteria were reported diagnoses of mental disorders, neurological disorders, intake of psychotropic medication and contraindications for MRI scans (e.g. pregnancy, metal implants).

Written informed consent was obtained from each participant before participation in the study. The Ethics Committee of the University of Graz approved the study (GZ 39/64/63 ex 2021/22), which was carried out following the current version of the Declaration of Helsinki. The study (sample size, design) was preregistered on the German Clinical Trial Register (DRKS 00030353, 05 October 2022). The study was conducted between October 2022 and September 2023.

### Images

The participants viewed 30 disgusting and 30 neutral images. The disgust pictures depicted elicitors of core disgust (e.g. disgusting animals such as snails and worms, rotten food and body secretions) and were taken from the International Affective Picture System (Lang *et al*., 2008) and a picture set of a previous placebo study (Schienle *et al*., 2017). The neutral images (also previously administered; Schienle *et al*., 2017) consisted of pixelated versions of the disgust images with a mosaic-like appearance (same color, luminance).

### Design

Participants were randomly allocated (random number table) to one of three groups: (i) oral intake of the OLP pill (1 cm-long capsule filled with 0.8 g dextrose) (OLP pill; *n* = 33), (ii) imagined intake of the OLP pill (Imaginary pill; *n* = 33) or (iii) passive viewing of the pictures without OLP instructions (PV; *n* = 33). The group-specific instructions were as follows:

*OLP pill*: ‘You will now receive a so-called open-label placebo that does not contain any active substances. This placebo is designed to help reduce emotional reactions to the negative images that will be shown to you. Scientific studies have demonstrated that placebos (even if you know they are placebos) can reduce negative emotions. You will now be given a glass of water and a placebo capsule for oral intake. This capsule is filled with sugar (dextrose). The placebo can support you so that the pictures are less negative for you.’

*Imaginary pill*: ‘It is of central importance that you understand: The belief that a placebo works is crucial for the placebo effect. The psychological component is critical. Thus, from a logical standpoint, it is not necessary to take an inactive substance (placebo) in order to be able to reduce negative emotions. Therefore, imagine that you are being supported by a placebo. It is an “imaginary placebo” that supports you so that the negative images that will be shown to you are less negative for you.’

*Passive viewing*: ‘You will now receive a pill to improve the MRI recordings. Functional magnetic resonance imaging (fMRI) is used to assess metabolic activity in the brain. It measures the oxygen content in the blood, which increases when neurons in the brain are activated. You will now receive a glass of water and a capsule for oral intake. This capsule is filled with dextrose (sugar). The substance slightly increases metabolism and thus improves the MRI signal-to-noise ratio.’ (A similar control instruction referring to the improvement of psychophysiological recordings has been used in an OLP study by Guevarra *et al*., 2020).

### Procedure

The participants were invited to an fMRI study on affective processing (no information about placebos was provided in the invitation). Before arriving at the fMRI lab, all participants filled in the brief Questionnaire for the Assessment of Disgust Propensity (QADP_brief, Schienle *et al*., 2020). In the lab, all groups viewed a presentation (14 PowerPoint slides with figures and text; no audio; fixed timing per slide: 30 s). Those in the OLP groups received information about the neurobiological effects of placebos with a focus on affective processing (findings from fMRI/EEG studies). The PV group received information about affective neuroscience (no placebo information). The presentations were comparable in the number of slides, figures and the number of words.

The participants of the OLP groups additionally evaluated their expectations concerning the effectiveness of the intervention (‘What do you think? How effective will the OLP be in reducing your negative emotional responses to the images?’ 1 = not effective; 9 = very effective). Then, the participants were brought to the fMRI lab (adjacent room). Here, the group-specific instructions were repeated verbally (OLP pill: ‘Please remember that the placebo you received can help you to reduce your negative emotional reactions to the images’; Imaginary pill: ‘Please remember that the imaginary placebo can help you to reduce your negative emotional reactions to the images’; PV: ‘Please remember to watch each image carefully for the entire duration of the presentation’).

The pictures were presented for 5 s each in blocks of three pictures of the same type (Disgust or Neutral). Then, a fixation cross was shown (variable interval: 2–4 s), which was followed by ratings for the intensity of experienced disgust (‘How intense was your experience of disgust?’; 1 = no disgust, 9 = very intense). Participants gave the ratings verbally using the intercom system. The picture presentation only continued when the rating was completed. After each rating, the trial ended with a 15-s resting period during which a fixation cross was shown. The experiment consisted of 10 disgust blocks (30 images) and 10 neutral blocks (30 images). The sequence of blocks was randomized with the only restriction that a maximum of two blocks of the same type could be shown consecutively.

At the end of the experiment, the OLP groups rated the perceived effectiveness of the treatment (‘How effective was the OLP in reducing your negative emotional responses to the images?’; 1 = not effective; 9 = very effective) and the perceived plausibility of the rationale (‘How plausible did you find the intervention? 1 = not at all; 9 = very).

The CONSORT diagram is depicted in Supplementary Figure S1.

### Statistical analysis of self-report data

Repeated-measures analyses of variance (ANOVAs) were conducted to test the effects of Group (OLP Pill, Imaginary Pill, Passive Viewing) and Picture Category (Disgust, Neutral) on the experience of disgust during the picture viewing. Ratings for the efficacy of the intervention were also analyzed via a repeated-measures ANOVA with the factors Group and Time (expected, perceived efficacy). For post-hoc analysis, results were considered statistically significant when the observed *P*-value was below the critical Bonferroni–Holm level (*P*_(Holm)_).

Before conducting these analyses, we checked whether the assumptions for the repeated-measures ANOVAs were met. While there were deviations in homoscedasticity and normality, we opted to adhere to the analysis plan, because an ANOVA is considered reasonably robust against violations of these assumptions, as long as each group has the same sample size (as in the present investigation).

An independent sample *t*-test compared plausibility ratings between two placebo groups. All statistical analyses were conducted with JAMOVI (version: 2.3.21).

### fMRI recordings and analysis

The MRI session was conducted with a 3 T scanner (Vida, Siemens, Erlangen, Germany) with a 64-channel head coil. Functional runs were acquired using a T2*-weighted multiband EPI protocol (number of slices: 58, interleaved, flip angle = 82°, slice thickness: 2.5 mm; slice spacing: 2.5 mm; TE = 0.03; TR =1800 ms; multi-band accel. factor = 2; acquisition matrix: 88; in-plane resolution = 2.5 × 2.5 × 2.5 mm). Structural images were obtained using a T1-weighted MPRAGE sequence (voxel size: 1 × 1 × 1 mm; acquisition matrix: 224, slice thickness: 1 mm, TE =0.00236, TR =1600 ms; flip angle = 9°). Preprocessing of the fMRI data was performed using *fMRIPrep* 22.0.1 (RRID:SCR_016216), which is based on *Nipype* 1.8.4 (Gorgolewski *et al*., 2011, 2018); RRID:SCR_002502; see Supplementary Material S1). The preprocessing of fMRI data included field homogeneity correction using an additional EPI with a different phase-encoding direction, skull stripping, anatomical image segmentation into gray matter, white matter and cerebrospinal fluid, motion correction, slice timing correction and normalization to a standard template in MNI space (voxel size: 2.5 mm isotropic). Subsequently, images were smoothed with a Gaussian kernel with a full width at half maximum of 8 mm (see S1 for a detailed description of the analysis procedure). First- and second-level analyses were carried out with SPM12 (7487) implemented in Matlab R2019b.

#### First-level analysis

Before conducting the first-level analysis, functional image quality metrics were evaluated using MRIQC (Esteban *et al*., 2017). The preprocessed images were then used for the first-level analyses, which involved convolving event-related responses for the image categories (Disgust, Neutral), and rating scales with the hemodynamic response function. We defined the contrast of interest as Disgust—Neutral. To account for motion-induced variance, we used the six motion parameters and their first derivative as regressors of no interest. Volumes with framewise displacement exceeding the predefined threshold of 0.5 mm were excluded from further analyses. No participant exceeded the predefined threshold of > 25% which would have led to exclution. The data were high-pass filtered at a frequency of 180 s and serial correlations were addressed by using an autoregressive AR(1) model.

### Second-level analysis

We compared the contrast Disgust—Neutral between groups (Imaginary pill *vs* OLP pill; Imaginary pill *vs* PV; OLP pill *vs* PV). Two regions of interest (ROIs: insula, pallidum) were selected based on a previous OLP study that administered the same disgust images (Schienle *et al*., 2023). In addition, brain activity in the amygdala, ventrolateral prefrontal cortex (VLPFC) and dorsolateral prefrontal cortex (DLPFC) was compared between the groups (according to the preregistration). The ROI masks were derived from the Harvard–Oxford probability atlas (with a threshold of 50%). Given the absence of ROI masks for the DLPFC and VLPFC in the Harvard–Oxford atlas, we used the inferior frontal gyrus as the VLPFC mask (Levy and Wagner 2011), while the middle and superior frontal gyrus formed the mask for the DLPFC (Yamagishi *et al*., 2016). Statistical significance was determined based on a family-wise error corrected (FWE) *P*-value for voxel-peaks that was <0.05. All results are small volume corrected. For effect sizes we report Cohen’s *d*.

We also performed a manipulation check to examine whether the observed activity patterns were related to disgust. When we included the disgust ratings for the images as a covariate of no interest in the model, statistically significant activation in brain regions central to disgust processing (insula, pallidum) disappeared.

## Results

### Questionnaire and socio-demographic data

The three groups did not differ in mean age [*F*(2,96) = 1.94, *P* = 0.149, *ɳ_p_*^2^ = 0.04; Imaginary Pill: M = 21.45 years, SD = 3.08; OLP Pill: M = 23.85 years, SD = 6.97; PV: M = 24.15 years, SD = 7.39] and in QADP scores [*F*(2,96) = 0.84, *P* = 0.433, *ɳ_p_*^2^ = 0.02; Imaginary Pill: M = 2.72, SD = 0.39; OLP Pill: M = 2.59, SD = 0.49; PV: M = 2.63, SD = 0.41].

### Disgust ratings

**Fig. 1.**
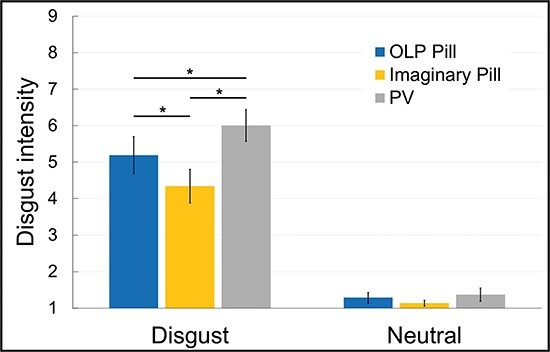
Mean ratings for disgust intensity for the two picture categories (disgust, neutral) in the three groups.

Note: Error bars indicate 95% confidence intervals. *P < 0.05.

The ANOVA for the disgust ratings revealed significant main effects for Group [*F*(2,96) = 13.30, *P* < 0.001, *ɳ_p_*^2^ = 0.22] and Picture Category [*F*(1,96) = 809.51, *P* < 0.001, *ɳ_p_*^2^ = 0.89], as well as a significant interaction Group × Picture Category [*F*(2,96) = 9.04, *P* < 0.001, *ɳ_p_*^2^ = 0.16]. Disgust pictures received higher disgust ratings than neutral pictures. Post-hoc *t*-tests indicated that both the OLP Pill group [*t*(64) = 2.41, *P* = 0.019, *d* = 0.59, *P*_(Holm)_ = 0.025] and the Imaginary Pill group [*t*(64) = 5.15, *P* <0.001, *d* = 1.27, *P*_(Holm)_ = 0.016] reported less intense disgust for the disgust pictures than the PV group. Moreover, the Imaginary Pill group reported less disgust than the OLP Pill group [*t*(64) = 2.42, *P* = 0.019, *d* = 0.60, *P*_(Holm)_ = 0.025] (see Figure 1; for violin plots see Supplementary Figure S2).

### Efficacy and plausibility of the interventions

#### Efficacy

The ANOVA revealed a significant main effect of Group [*F*(1,64) = 87.80, *P* < 0.001, *ɳ_p_*^2^ = 0.58], a significant main effect of time [*F*(1,64) = 6.67, *P* = 0.012, *ɳ_p_*^2^ = 0.09] and a significant interaction Group × Time (*F*(1,64) = 24.82, *p* < 0.001, *ɳ_p_*^2^ = 0.28). The Imaginary Pill group gave higher ratings for efficacy than the OLP Pill group before and after the experiment [expected: *t*(64) = 3.87, *P* < 0.001, *d* = 0.95, *P*_(Holm)_ = 0.016; perceived: *t*(64) = 11.4, *P* < 0.001, *d* = 2.80, *P*_(Holm)_ = 0.016]. In the Imaginary Pill group, perceived efficacy was higher than expected efficacy [*t*(32) = 6.38, *P* < 0.001, *d* = 1.11, *P*_(Holm)_ = 0.016]. Expected and perceived efficacy did not differ in the OLP pill group [*t*(32) = 1.49, *P* = 0.146, *d* = 0.26] (see Table 1). Due to the hybrid interaction, the main effect of time could not be interpreted.

**Table 1.** Ratings for efficacy (expected, perceived) and plausibility of the intervention

	OLP pill M (SD)	Imaginary pill M (SD)
	[95% CI]	[95% CI]
	Ratings [1.9]	
Expected efficacy	5.39 (1.27)	6.48 (1.00)
	[4.94, 5.79]	[6.15, 6.85]
Perceived efficacy	5.00 (1.23)	7.73 (0.63)
	[4.61, 5.42]	[7.52, 7.94]
Plausibility	6.45 (2.09)	8.21 (0.93)
	[5.73, 7.12]	[7.91, 8.52]

#### Plausibility

The Imaginary Pill group gave higher plausibility ratings for the intervention than the OLP Pill group [*t*(64) = 4.41, *P* < 0.001, *d* = 1.09] (see Table 1).

### Brain activity

**Fig. 2.**
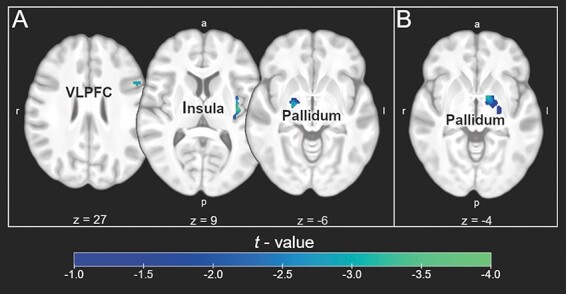
Reduced activation (disgust—neutral) in the imaginary pill group relative to PV (A) and the OLP pill group (B).

Note: VLPFC: Ventrolateral Prefrontal Cortex.

Relative to the OLP pill, the imaginary pill reduced activity in the pallidum (contrast: Disgust—Neutral). The Imaginary Pill group was characterized by lower activity in the insula, pallidum and VLPFC than the PV group (see Figure 2 and Table 2). No significant differences in ROI activation were found when comparing the OLP pill with PV.

**Table 2.** Group comparison of brain activity (contrast: Disgust—Neutral)

Region of interest	H	*x*	*y*	*z*	T	p_FWE-corr	Cluster size	Cohen’s *d*
Passive Viewing—Imaginary pill								
VLPFC	L	−49	15	27	3.04	0.0344	59	0.76
Insula	L	−39	−13	9	3.45	0.0261	86	0.86
Pallidum	R	21	−10	−6	3.01	0.0335	41	0.75
OLP pill—Imaginary pill								
Pallidum	L	−14	3	−4	3.19	0.0215	55	0.78

Note: VLPFC: Ventrolateral Prefrontal Cortex; H: hemisphere; x, y,z: MNI coordinates; *P* corrected for family-wise error; cluster size: number of voxels; cluster-building threshold (uncorrected): 0.05.

The analysis did not reveal any whole-brain findings that were statistically significant. For significant results of the within-group comparisons, see Supplementary Table S1.

## Discussion

The present fMRI study compared the effects of an OLP pill and an imaginary pill for reducing visually induced disgust. The results showed that both placebo interventions lowered the intensity of experienced revulsion compared to the PV of the disgust pictures. This finding is in line with a recent meta-analysis that revealed significant effects of OLP interventions (with actual OLP intake) on different forms of self-reported emotional distress (Spille *et al*., 2023). Furthermore, we found similar effects as Buergler *et al*. (2023a), who demonstrated that an imaginary pill was able to reduce a negative affective state (test anxiety) in healthy university students. In that study, the efficacy of the administered pill and imaginary pill was comparable. This was different in the present investigation, where the imaginary pill was perceived as more effective than the OLP pill. The ‘imaginary placebo’ was associated with lower disgust ratings and higher ratings for expected as well as perceived efficacy. Interestingly, in the Imaginary Pill group, efficacy was rated more highly after the experiment than before (perceived efficacy > expected efficacy). The participants seemed positively surprised about this type of intervention. Moreover, participants gave higher plausibility ratings for the Imaginary Pill intervention than for the OLP pill. Taken together, the self-report data question the common assumption that the actual intake of the pill is a crucial factor for OLP responses (Kaptchuk *et al*., 2010). The present findings reveal the power of the purely psychological component of a placebo (Buergler *et al*., 2023a). In line, previous studies have already demonstrated that placebo effects can be realized without a physical treatment component (e.g. Gaab *et al*., 2019).

The analysis of the fMRI data showed that the imaginary pill was associated with less activity in the pallidum compared to the OLP pill. Moreover, relative to the PV of the disgusting images, the imaginary pill reduced insula activity. Previous research has consistently demonstrated the involvement of both the pallidum and the insula in the processing of disgust (Phillips *et al*., 1998, 2000; Sprengelmeyer *et al*., 1998; Wicker *et al*., 2003; Calder *et al*., 2007; Ho and Berridge, 2014).

The pallidum is an important structure for the processing of the hedonic value of stimuli. It is central to the acquisition of disgust responses (e.g. food aversion) as demonstrated in studies with both humans and animals (Khan *et al*., 2020; Morales and Berridge, 2020). The insula is part of a salience network that is involved in the generation of affective states, including disgust (Uddin, 2015). A successful down-regulation of disgust feelings via different emotion regulation strategies (e.g. cognitive reappraisal, deceptive placebo treatment) has been associated with changes in insula activity before (Goldin *et al*., 2008; Schienle *et al*., 2014).

Finally, the imaginary pill was associated with less VLPFC activity than PV. Generally, the VLPFC plays a significant role in emotion regulation (Goldin *et al*., 2008). For example, cognitive reappraisal (CR), which involves reinterpreting or reframing the meaning of a situation in order to change one’s emotional response, has been associated with increased VLPFC activity (Goldin *et al*., 2008; Schienle *et al*., 2023). CR is an effortful regulation strategy with a specific goal-directed task [e.g. the participants are instructed to take an objective/ professional perspective while being exposed to negative stimuli (Goldin *et al*., 2008)]. In contrast, in the present study, the participants received a supporting ‘imaginary placebo’ without a specific task. This type of placebo intervention may require less cognitive effort than CR. Therefore, a future investigation should assess the subjective effort related to emotion control that is associated with different OLP interventions.

In addition, based on the results of their meta-analysis of brain imaging data concerning cognitive emotion control, Kohn *et al*. (2014) concluded that the VLPFC may not necessarily reflect the regulatory process per se, but may detect salience and the need to regulate an affective state. Notably, the OLP Pill group did not exhibit significant VLPFC activity during disgust processing when compared with the PV group—a finding previously identified in a study by Schienle *et al*. (2023). This discrepancy suggests potential individual differences in cognitive processes (appraisals) associated with OLP treatment. According to Ashar *et al*. (2017) placebo-related appraisals are complex mental representations (constructed interpretations) encompassing precognitive associations, memories, expectations, goals and interoceptive components. The involvement of these components likely varies among individuals, leading to the recruitment of different brain regions. Within this context, it is essential to note that the placebo effects do not necessarily require VLPFC recruitment. For instance, Jensen *et al*. (2015) demonstrated that placebo cues can be processed even subconsciously, which was associated with subcortical activation representing a reward-related signal rather than cognitive control.

Two additional ROIs (amygdala, DLPFC), selected based on previous research on deceptive placebos, also exhibited no significant activation in the group comparisons. To comprehend the absence of these effects, a direct comparison of neural responses to deceptive *vs* nondeceptive placebo treatment on disgust regulation would be beneficial.

The present study has several limitations. First, the study sample included only females, thus limiting the generalizability of the results. Second, we used disgust-eliciting pictures. It remains to be seen whether the observed results would generalize to other types of emotional stimuli. Third, the group sizes were relatively small (OLP Pill/ Imaginary Pill/ Passive Viewing; *n* = 33 per group) as compared to an earlier OLP-fMRI study (Schienle *et al*., 2023; *n* = 50 per group). However, (Schaefer *et al.*, 2023) reported differences in brain activity when comparing 23 participants who received an OLP for reducing emotional distress with 21 who did not.

Fourth, the current experimental design did not include the assessment of qualitative data, such as patients’ explanations of how the OLPs exerted their effects. For instance, in a study by Haas *et al*. (2022), a mixed-design approach compared self-reports of participants who received deceptive *vs* nondeceptive placebos. OLP participants engaged more in reflections on their treatment and were significantly less likely to attribute improvement to the OLP compared to those with deceptive placebos.

Finally, a potential problem with the current study is the selective focus on healthy volunteers. There could be fundamental differences in the mechanisms contributing to the placebo effect, such as motivation and expectation, between individuals with and without mental/somatic disorders (Spille *et al*., 2023). It is plausible that patients’ placebo responses may be mediated via different neural pathways that are more related to these motivational processes (e.g. hope in change). Additionally, existing evidence has already demonstrated that OLP effects tend to be more pronounced in clinical as opposed to nonclinical samples (Buergler *et al*., 2023b).

In summary, the present fMRI study is the first to compare the effects of different forms of OLP administration/rationales. It demonstrated that placebo effects can be harnessed without the use of a physical treatment component. The intervention with the imaginary pill was a more effective and plausible alternative to the traditional OLP approach for alleviating feelings of disgust. These findings have important implications for the development of new OLP-based interventions for nonclinical as well as clinical contexts.

## Supplementary Material

nsae021_Supp

## Data Availability

Data are available from the first author upon request.

## References

[R1] Ashar Y.K., Chang L.J., Wager T.D. (2017). Brain mechanisms of the placebo effect: an affective appraisal account. *Annual Review of Clinical Psychology*, 13(1), 73–98. 10.1146/annurev-clinpsy-021815-093015 28375723

[R2] Buergler S., Sezer D., Bagge N., et al. (2023a). Imaginary pills and open-label placebos can reduce test anxiety by means of placebo mechanisms. *Scientific Reports*, 13, 2624. 10.1038/s41598-023-29624-7 PMC9926426 36788309

[R3] Buergler S., Sezer D., Gaab J., Locher C. (2023b). The roles of expectation, comparator, administration route, and population in open-label placebo effects: A network meta-analysis. *Scientific Reports*, 13(1), 11827. 10.1038/s41598-023-39123-4 PMC10363169 37481686

[R4] Calder A.J., Beaver J.D., Davis M.H., van Ditzhuijzen J., Keane J., Lawrence A.D. (2007). Disgust sensitivity predicts the insula and pallidal response to pictures of disgusting foods In. *The European Journal of Neuroscience*, 5, 3422–8. 10.1111/j.1460-9568.2007.05604.x 17553011

[R5] Colloca L., Howick J. (2018). Placebos without deception. outcomes mechanisms and ethics. *International Review of Neurobiology*, 138, 219–40. 29681327 10.1016/bs.irn.2018.01.005 PMC5918690

[R6] Enck P., Klosterhalfen S. (2019). Does sex/gender play a role in placebo and nocebo effects? Conflicting evidence from clinical trials and experimental studies in. *Frontiers in Neuroscience*., 13, 160. 10.3389/fnins.2019.00160 PMC6409330 30886569

[R7] Esteban O., Birman D., Schaer M., Koyejo O.O., Poldrack R.A., Gorgolewski K.J. (2017). MRIQC. Advancing the automatic prediction of image quality in MRI from unseen sites In. *PloS One*, 12(9), e0184661. 10.1371/journal.pone.0184661 PMC5612458 28945803

[R8] Gaab J., Kossowsky J., Ehlert U., Locher C. (2019). Effects and components of placebos with a psychological treatment rationale—Three randomized-controlled studies. *Scientic Reports*, 9, 1421. 10.1038/s41598-018-37945-1 PMC6363794 30723231

[R9] Goldin P.R., McRae K., Ramel W., Gross J.J. (2008). The neural bases of emotion regulation. Reappraisal and suppression of negative emotion. *Biological Psychiatry*, 63(6), 577–86. 17888411 10.1016/j.biopsych.2007.05.031 PMC2483789

[R10] Gorgolewski K., Burns C.D., Madison C., et al. (2011). Nipype. A flexible lightweight and extensible neuroimaging data processing framework in python In. *Frontiers in Neuroinformatics*, 5, 13. 10.3389/fninf.2011.00013 PMC3159964 21897815

[R11] Gorgolewski K.J., Esteba O., Markiewicz C.J., et al. (2018). Nipype [Software].

[R12] Guevarra D.A., Moser J.S., Wager T.D., Kross E. (2020). Placebos without deception reduce self-report and neural measures of emotional distress. *Nature Communications*, 11(1), 3785. 10.1038/s41467-020-17654-y PMC7391658 32728026

[R13] Haas J.W., Ongaro G., Jacobson E., et al. (2022). Patients’ experiences treated with open-label placebo versus double-blind placebo: a mixed methods qualitative study. *BMC Psychology*, 10(1), 20. 10.1186/s40359-022-00731-w PMC8815135 35120572

[R14] Ho C.Y., Berridge K.C. (2014). Excessive disgust caused by brain lesions or temporary inactivations. mapping hotspots of the nucleus accumbens and ventral pallidum In. *European Journal of Neuroscience*, 40(10), S 3556–3572. 10.1111/ejn.12720 PMC4236281 25229197

[R15] Jensen K.B., Kaptchuk T.J., Chen X., et al. (2015). A neural mechanism for nonconscious activation of conditioned placebo and nocebo responses. *Cerebral Cortex*, 25(10), 3903–10. 25452576 10.1093/cercor/bhu275 PMC4585522

[R16] Kaptchuk T.J., Friedlander E., Kelley J.M., et al. (2010). Placebos without deception. a randomized controlled trial in irritable bowel syndrome. *PloS One*, 5(12), e15591. 10.1371/journal.pone.0015591 PMC3008733 21203519

[R17] Khan H.A., Urstadt K.R., Mostovoi N.A., Berridge K.C. (2020). Mapping excessive “disgust” in the brain. Ventral pallidum inactivation recruits distributed circuitry to make sweetness “disgusting”. *Cognitive, Affective, & Behavioral Neuroscience*, 20(1), 141–59. 10.3758/s13415-019-00758-4 PMC7018599 31836960

[R18] Koban L., Kross E., Woo C.W., Ruzic L., Wager T.D. (2017). Frontal-brainstem pathways mediating placebo effects on social rejection In. *The Journal of Neuroscience*, 37(13), S 3621–3631. 10.1523/JNEUROSCI.2658-16.2017 PMC5373138 28264983

[R19] Kohn N., Eickhoff S.B., Scheller M., Laird A.R., Fox P.T., Habel U. (2014). Neural network of cognitive emotion regulation—an ALE meta-analysis and MACM analysis In. *NeuroImage*, 87, 345–55. 24220041 10.1016/j.neuroimage.2013.11.001 PMC4801480

[R20] Lang P.J., Bradley M.M., Cuthbert B.N. (2008). International affective picture system (IAPS). Affective ratings of pictures and instruction manual Gainesville FL. University of Florida.

[R21] Levy B.J., Wagner A.D. (2011). Cognitive control and right ventrolateral prefrontal cortex: Refexive reorienting, motor inhibition, and action updating. *Annals of the New York*, 1224(1), 40–62. 10.1111/j.1749-6632.2011.05958.x PMC3079823 21486295

[R22] Meyer B., Yuen K.S.L., Ertl M., et al. (2015). Neural mechanisms of placebo anxiolysis. *The Journal of Neuroscience*, 35(19), S 7365–7373. 25972166 10.1523/JNEUROSCI.4793-14.2015 PMC6705432

[R23] Meyer B., Yuen K.S.L., Saase V., Kalisch R. (2019). The functional role of large-scale brain network coordination in placebo-induced anxiolysis. *Cerebral Cortex*, 29(8), 3201–10. 30124792 10.1093/cercor/bhy188

[R24] Morales I., Berridge K.C. (2020). ‘Liking’ and ‘wanting’ in eating and food reward. Brain mechanisms and clinical implications. *Physiology and Behavior*, 227, 113152. 10.1016/j.physbeh.2020.113152 PMC7655589 32846152

[R25] Petrovic P., Dietrich T., Fransson P., Andersson J., Carlsson K., Ingvar M. (2005). Placebo in emotional processing—induced expectations of anxiety relief activate a generalized modulatory network In. *Neuron*, 46(6), 957–69. 15953423 10.1016/j.neuron.2005.05.023

[R26] Phillips M.L., Young A.W., Scott S.K., Calder A.J., Andrew C., Giampietro V. (1998). Neural responses to facial and vocal expressions of fear and disgust In. *Proceedings of the Royal Society of London Series B Biological Sciences*, 265(1408), 1809–17. 10.1098/rspb.1998.0506 PMC1689379 9802236

[R27] Phillips M.L., Marks I.M., Senior C., Lythgoe D., O’Dwyer A.M., Meehan O. (2000). A differential neural response in obsessive-compulsive disorder patients with washing compared with checking symptoms to disgust In. *Psychological Medicine*, 30(5), 1037–50. 12027041 10.1017/s0033291799002652

[R28] Schaefer M., Kühnel A., Schweitzer F., Enge S., Gärtner M. (2023). Neural underpinnings of open-label placebo effects in emotional distress. *Neuropsychopharmacology*, 48(3), S 560–566. 36456814 10.1038/s41386-022-01501-3 PMC9852452

[R29] Schienle A., Übel S., Schöngaßner F., Ille R., Scharmüller W. (2014). Disgust regulation via placebo. *An fMRI Study Social Cognitive and Affective Neuroscience*, 9(7), 985–90. 23868896 10.1093/scan/nst072 PMC4090961

[R30] Schienle A., Übel S., Wabnegger A. (2017). When opposites lead to the same. a direct comparison of explicit and implicit disgust regulation via fMRI In. *Social Cognitive & Affective Neuroscience*, 12(3), S 445–451. 27665000 10.1093/scan/nsw144 PMC5390737

[R31] Schienle A., Zorjan S., Wabnegger A. (2020). A brief measure of disgust propensity. *Current Psychology*, 41(6), 3687–93.

[R32] Schienle A., Kogler W., Wabnegger A. (2023). A randomized trial that compared brain activity efficacy and plausibility of open-label placebo treatment and cognitive repappraisal for reducing emotional distress. *Scientific Reports*, 13, 13998. 10.1038/s41598-023-39806-y PMC10460441 37634020

[R33] Spille L., Fendel J.C., Seuling P., Göritz A.S., Schmidt S. (2023). Open-label placebos—a systematic review and meta-analysis of experimental studies with non-clinical samples. *Scientific Reports*, 13(1). 10.1038/s41598-023-30362-z PMC9985604 36871028

[R34] Sprengelmeyer R., Rausch M., Eysel U.T., Przuntek H. (1998). Neural structures associated with recognition of facial expressions of basic emotions In. *Proceedings of the Royal Society of London Series B Biological Sciences*, 265(1409), 1927–31. 10.1098/rspb.1998.0522 PMC1689486 9821359

[R35] Uddin L.Q. (2015). Salience processing and insular cortical function and dysfunction. *Nature Reviews, Neuroscience*, 16(1), S 55–61. 10.1038/nrn3857 25406711

[R36] Wicker B., Keysers C., Plailly J., Royet J.P., Gallese V., Rizzolatti G. (2003). Both of us disgusted in My insula. the common neural basis of seeing and feeling disgust. *Neuron*, 40(3), S 655–664. 14642287 10.1016/s0896-6273(03)00679-2

[R37] Yamagishi T., Takagishi H., Fermin A.D.S.R., Kanai R., Li Y., Matsumoto Y. (2016). Cortical thickness of the dorsolateral prefrontal cortex predicts strategic choices in economic games. *Proceedings of the National Academy of Science*, 113(20), 5582–7. 10.1073/pnas.1523940113 PMC4878491 27140622

