# Supplementary material for: The pill you don’t have to take that is still effective: neural correlates of imaginary placebo intake for regulating disgust
Source: Soc Cogn Affect Neurosci. 2024 Mar 7;19(1):nsae021. doi: 10.1093/scan/nsae021 (PMC11227952; doi:10.1093/scan/nsae021)

**Supplementary Material**

**Supplementary Material S1: Preprocessing of fMRI data**

Results included in this manuscript come from preprocessing performed using *fMRIPrep* 22.0.1 (Esteban et al. (2018a); Esteban et al. (2018b); RRID:SCR_016216), which is based on *Nipype* 1.8.4 (Gorgolewski et al. (2011); K. J. Gorgolewski et al. (2018); RRID:SCR_002502). Smoothing, first and second-level analyses were carried out with SPM12 (7487) implemented in Matlab R2019b.

The following paragraph is an exact copy of the output of fmriprep. For the current analyses, we used the motion parameters and their first derivative as well as framewise displacement (> 0.5 mm) as covariates of no interest.

Preprocessing of B_0_ inhomogeneity mappings: A total of 1 fieldmaps were found available within the input BIDS structure for this particular subject. A B0-nonuniformity map (or field map) was estimated based on two (or more) echo-planar imaging (EPI) references with top up (Andersson, Skare, and Ashburner (2003); FSL 6.0.5.1:57b01774).

*Anatomical data preprocessing*: A total of 1 T1-weighted (T1w) images were found within the input BIDS dataset. The T1-weighted (T1w) image was corrected for intensity non-uniformity (INU) with N4BiasFieldCorrection (Tustison et al. 2010), distributed with ANTs 2.3.3 (Avants et al. 2008, RRID:SCR_004757), and used as T1w-reference throughout the workflow. The T1w-reference was then skull-stripped with a *Nipype* implementation of the antsBrainExtraction.sh workflow (from ANTs), using OASIS30ANTs as target template. Brain tissue segmentation of cerebrospinal fluid (CSF), white matter (WM), and gray matter (GM) was performed on the brain-extracted T1w using fast (FSL 6.0.5.1:57b01774, RRID:SCR_002823, Zhang, Brady, and Smith 2001). Volume-based spatial normalization to one standard space (MNI152NLin2009cAsym) was performed through nonlinear registration with antsRegistration (ANTs 2.3.3), using brain-extracted versions of both T1w reference and the T1w template. The following template was selected for spatial normalization: *ICBM 152 Nonlinear Asymmetrical template version 2009c* [Fonov et al. (2009), RRID:SCR_008796; TemplateFlow ID: MNI152NLin2009cAsym].

*Functional data preprocessing*: For each of the 1 BOLD runs found per subject (across all tasks and sessions), the following preprocessing was performed. First, a reference volume and its skull-stripped version were generated using a custom methodology of *fMRIPrep*. Head-motion parameters for the BOLD reference (transformation matrices, and six corresponding rotation and translation parameters) are estimated before any spatiotemporal filtering using mcflirt (FSL 6.0.5.1:57b01774, Jenkinson et al. 2002). The estimated *fieldmap* was then aligned with rigid registration to the target EPI (echo-planar imaging) reference run. The field coefficients were mapped onto the reference EPI using the transform. BOLD runs were slice-time corrected to 0.859s (0.5 of slice acquisition range 0s-1.72s) using 3dTshift from AFNI (Cox and Hyde 1997, RRID:SCR_005927). The BOLD reference was then co-registered to the T1w reference using mri_coreg (FreeSurfer) followed by flirt (FSL 6.0.5.1:57b01774, Jenkinson and Smith 2001) with the boundary-based registration (Greve and Fischl 2009) cost-function. Co-registration was configured with six degrees of freedom. Several confounding time series were calculated based on the *preprocessed BOLD*: framewise displacement (FD), DVARS, and three region-wise global signals. FD was computed using two formulations following Power (absolute sum of relative motions, Power et al. (2014)) and Jenkinson (relative root mean square displacement between affines, Jenkinson et al. (2002)). FD and DVARS are calculated for each functional run, both using their implementations in *Nipype* (following the definitions by Power et al. 2014). The three global signals are extracted within the CSF, the WM, and the whole-brain masks. Additionally, a set of physiological regressors were extracted to allow for component-based noise correction (*CompCor*, Behzadi et al. 2007). Principal components are estimated after high-pass filtering the *preprocessed BOLD* time-series (using a discrete cosine filter with 128s cut-off) for the two *CompCor* variants: temporal (tCompCor) and anatomical (aCompCor). tCompCor components are then calculated from the top 2% variable voxels within the brain mask. For aCompCor, three probabilistic masks (CSF, WM and combined CSF+WM) are generated in anatomical space. The implementation differs from that of Behzadi et al. in that instead of eroding the masks by 2 pixels on BOLD space, a mask of pixels that likely contain a volume fraction of GM is subtracted from the aCompCor masks. This mask is obtained by thresholding the corresponding partial volume map at 0.05, and it ensures components are not extracted from voxels containing a minimal fraction of GM. Finally, these masks are resampled into BOLD space and binarized by thresholding at 0.99 (as in the original implementation). Components are also calculated separately within the WM and CSF masks. For each CompCor decomposition, the *k* components with the largest singular values are retained, such that the retained components’ time series are sufficient to explain 50 percent of variance across the nuisance mask (CSF, WM, combined, or temporal). The remaining components are dropped from consideration. The head-motion estimates calculated in the correction step were also placed within the corresponding confounds file. The confound time series derived from head motion estimates and global signals were expanded with the inclusion of temporal derivatives and quadratic terms for each (Satterthwaite et al. 2013). Frames that exceeded a threshold of 0.5 mm FD or 1.5 standardized DVARS were annotated as motion outliers. Additional nuisance timeseries are calculated by means of principal components analysis of the signal found within a thin band (*crown*) of voxels around the edge of the brain, as proposed by (Patriat, Reynolds, and Birn 2017). The BOLD time series were resampled into standard space, generating a *preprocessed BOLD run in MNI152NLin2009cAsym space*. First, a reference volume and its skull-stripped version were generated using a custom methodology of *fMRIPrep*. All resamplings can be performed with *a single interpolation step* by composing all the pertinent transformations (i.e. head-motion transform matrices, susceptibility distortion correction when available, and co-registrations to anatomical and output spaces). Gridded (volumetric) resamplings were performed using antsApplyTransforms (ANTs), configured with Lanczos interpolation to minimize the smoothing effects of other kernels (Lanczos 1964). Non-gridded (surface) resamplings were performed using mri_vol2surf (FreeSurfer). Finally, functional images were smoothed with a Gaussian full width at half maximum of 8mm.

**References**

Abraham, A., Pedregosa F., Eickenberg M., Gervais P., Mueller A., Kossaifi J., et al. (2014). “Machine Learning for Neuroimaging with Scikit-Learn.” *Frontiers in Neuroinformatics* 8. <https://doi.org/10.3389/fninf.2014.00014>.

Andersson, J.L.R., Skare S., & Ashburner J. (2003). “How to Correct Susceptibility Distortions in Spin-Echo Echo-Planar Images: Application to Diffusion Tensor Imaging.” *NeuroImage* 20 (2): 870–88. <https://doi.org/10.1016/S1053-8119(03)00336-7>.

Avants, B.B., Epstein C.L., Grossman M., & Gee J. C.. (2008). “Symmetric Diffeomorphic Image Registration with Cross-Correlation: Evaluating Automated Labeling of Elderly and Neurodegenerative Brain.” *Medical Image Analysis* 12 (1): 26–41. <https://doi.org/10.1016/j.media.2007.06.004>.

Behzadi, Y., Restom, K., Liau, J., & Liu, T.T. (2007). A component based noise correction method (CompCor) for BOLD and perfusion based fMRI. *NeuroImage*, *37*(1), 90–101. https://doi.org/10.1016/j.neuroimage.2007.04.042

Cox, R.W., & Hyde, J.S. (1997). Software tools for analysis and visualization of fMRI data. *NMR in biomedicine*, *10*(4-5), 171–178. https://doi.org/10.1002/(sici)1099-1492(199706/08)10:4/5<171::aid-nbm453>3.0.co;2-l

Esteban O., Birman D., Schaer M., Koyejo O.O., Poldrack R. A. & Gorgolewski K.J. (2017); *MRIQC: Advancing the Automatic Prediction of Image Quality in MRI from Unseen Sites*; PLOS ONE 12(9):e0184661; doi:[10.1371/journal.pone.0184661](https://doi.org/10.1371/journal.pone.0184661).

Esteban, O., Blair R., Markiewicz, C.J., Berleant, S.L., Moodie, C., Ma, F. et al. (2018a). “fMRIPrep 22.0.1.” *Software*. <https://doi.org/10.5281/zenodo.852659>.

Esteban, O., Markiewicz, C., Blair, R.W., Moodie, C., Isik, A.I., Erramuzpe A.A., et al. (2018b). “fMRIPrep: A Robust Preprocessing Pipeline for Functional MRI.” *Nature Methods*. <https://doi.org/10.1038/s41592-018-0235-4>.

Fonov, V.S., Evans, A.C., McKinstry, R.C., Almli, C.R., & Collins, D.L. (2009). “Unbiased Nonlinear Average Age-Appropriate Brain Templates from Birth to Adulthood.” *NeuroImage* 47, Supplement 1: S102. <https://doi.org/10.1016/S1053-8119(09)70884-5>.

Gorgolewski, K., Burns, C.D., Madison,C., Clark, D., Halchenko, Y.O., Waskom,M.L., et al. (2011). “Nipype: A Flexible, Lightweight and Extensible Neuroimaging Data Processing Framework in Python.” *Frontiers in Neuroinformatics* 5: 13. <https://doi.org/10.3389/fninf.2011.00013>.

Gorgolewski, K., Esteban, O., Markiewicz, C.J., Ziegler, E., Ellis, D.G., Notter, M.P. et al. (2018). “Nipype.” *Software*. <https://doi.org/10.5281/zenodo.596855>.

Greve, D. N., & Fischl, B. (2009). “Accurate and Robust Brain Image Alignment Using Boundary-Based Registration.” *NeuroImage* 48 (1): 63–72. <https://doi.org/10.1016/j.neuroimage.2009.06.060>.

Jenkinson, M., & Smith, S. (2001). “A Global Optimisation Method for Robust Affine Registration of Brain Images.” *Medical Image Analysis* 5 (2): 143–56. <https://doi.org/10.1016/S1361-8415(01)00036-6>.

Jenkinson, M., Bannister, P., Brady, M. & Smith, S. (2002). “Improved Optimization for the Robust and Accurate Linear Registration and Motion Correction of Brain Images.” *NeuroImage* 17 (2): 825–41. <https://doi.org/10.1006/nimg.2002.1132>.

Lanczos, C. (1964). “Evaluation of Noisy Data.” *Journal of the Society for Industrial and Applied Mathematics Series B Numerical Analysis* 1 (1): 76–85. <https://doi.org/10.1137/0701007>.

Patriat, R., Reynolds, R.C., & Birn, R.M. (2017). “An Improved Model of Motion-Related Signal Changes in fMRI.” *NeuroImage* 144, Part A (January): 74–82. <https://doi.org/10.1016/j.neuroimage.2016.08.051>.

Power, J.D., Mitra, A., Laumann, T.O., Snyder, A.Z., Schlaggar, B.L. & Petersen, S.E. (2014). “Methods to Detect, Characterize, and Remove Motion Artifact in Resting State fMRI.” *NeuroImage* 84 (Supplement C): 320–41. <https://doi.org/10.1016/j.neuroimage.2013.08.048>.

Satterthwaite, T.D., Elliott, M.A., Gerraty, R.T., Ruparel, K., Loughead, J., Calkins, M.E. et al. (2013). “An improved framework for confound regression and filtering for control of motion artifact in the preprocessing of resting-state functional connectivity data.” *NeuroImage* 64 (1): 240–56. <https://doi.org/10.1016/j.neuroimage.2012.08.052>.

Tustison, N.J., Avants, B.B., Cook, P.A., Zheng, Y., Egan, A., Yushkevich, P.A. et al. (2010). “N4itk: Improved N3 Bias Correction.” *IEEE Transactions on Medical Imaging* 29 (6): 1310–20. <https://doi.org/10.1109/TMI.2010.2046908>.

Zhang, Y., Brady, M. & Smith, S. (2001). “Segmentation of Brain MR Images Through a Hidden Markov Random Field Model and the Expectation-Maximization Algorithm.” *IEEE Transactions on Medical Imaging* 20 (1): 45–57. <https://doi.org/10.1109/42.906424>.

**Supplementary Table S1:** Brain Activity (Contrast: Disgust - Neutral) Within The Three Groups

| **ROI** | |  | **H** | **x** | **y** | **Z** | **T** | **p_FWE-corr** | **Cluster Size** | **Cohen’s d** |
| --- | --- | --- | --- | --- | --- | --- | --- | --- | --- | --- |
|  | **Passive Viewing** | | | | | | | | |  |
| VLPFC | |  | L | -52 | 13 | 27 | 7.13 | <.001 | 137 | 2.52 |
| VLPFC | |  | R | 51 | 15 | 22 | 4.18 | .002 | 82 | 1.48 |
| Insula | |  | L | -34 | 13 | 4 | 9.79 | <.001 | 222 | 3.46 |
| Insula | |  | R | 36 | 13 | -14 | 10.02 | <.001 | 248 | 3.54 |
| Pallidum | |  | L | -22 | -10 | -6 | 7.95 | <.001 | 115 | 2.81 |
| Pallidum | |  | R | 14 | -5 | -6 | 8.33 | <.001 | 115 | 2.95 |
| Amygdala | |  | L | -24 | -5 | -14 | 15.60 | <.001 | 106 | 5.52 |
| Amygdala | |  | R | 21 | -3 | -14 | 14.21 | <.001 | 150 | 5.02 |
|  | **Imaginary Pill** | | | | | | | | |  |
| VLPFC | |  | L | -52 | 18 | 2 | 6.14 | <.001 | 137 | 2.17 |
| VLPFC | |  | R | 51 | 15 | 27 | 3.28 | .020 | 14 | 1.16 |
| Insula | |  | L | -34 | 8 | -14 | 12.42 | <.001 | 202 | 4.39 |
| Insula | |  | R | 33.5 | 10 | -16 | 9.05 | <.001 | 240 | 3.20 |
| Pallidum | |  | L | -12 | 3 | -1 | 6.94 | <.001 | 114 | 2.45 |
| Pallidum | |  | R | -14 | 5 | -1 | 6.87 | <.001 | 115 | 1.98 |
| Amygdala | |  | L | -22 | -5 | 14 | 13.72 | <.001 | 107 | 0.96 |
| Amygdala | |  | R | 24 | 3 | -19 | 11.82 | <.001 | 150 | 4.18 |
|  | **OLP Pill** | | | | | | | | |  |
| VLPFC | |  | L | -52 | 15 | -1 | 7.21 | <.001 | 136 | 2.55 |
| VLPFC | |  | R | 51 | 15 | 24 | 4.39 | .001 | 61 | 1.55 |
| Insula | |  | L | -39 | 10 | -6 | 11.43 | <.001 | 213 | 4.04 |
| Insula | |  | R | 36 | 8 | -14 | 10.18 | <.001 | 252 | 3.60 |
| Pallidum | |  | L | -12 | 3 | -1 | 10.01 | <.001 | 118 | 3.54 |
| Pallidum | |  | R | 16 | 0 | -6 | 7.55 | <.001 | 114 | 2.67 |
| Amygdala | |  | L | -24 | -5 | -14 | 14.90 | <.001 | 103 | 5.27 |
| Amygdala | |  | R | 24 | -5 | -14 | 14.82 | <.001 | 142 | 5.24 |

Note: Ventrolateral Prefrontal Cortex (VLPFC); Cluster-Building Threshold (Uncorrected): 0.05

**Supplemetary Figure S1: CONSORT Diagram**

**
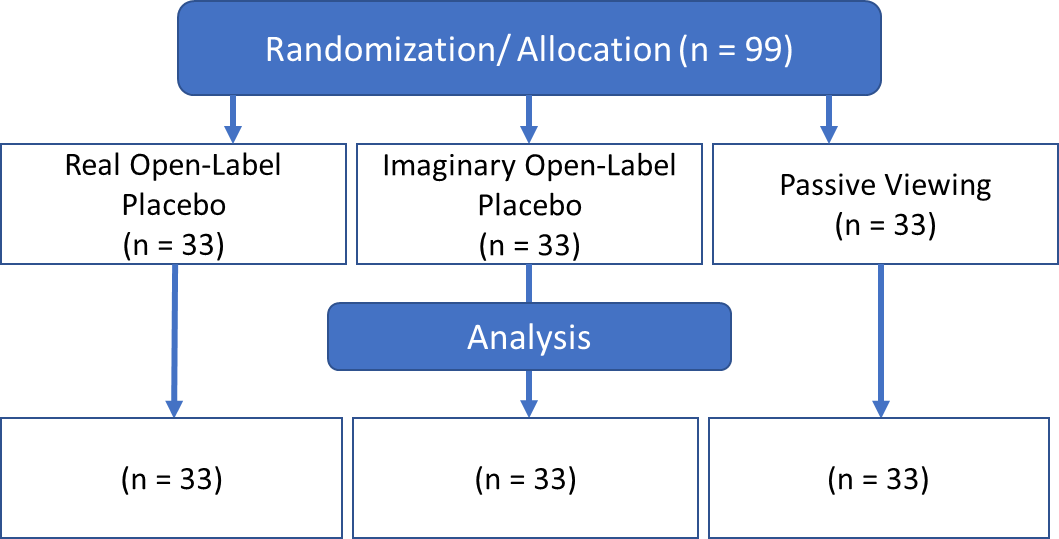
**

**Supplementary Figure S2: Violin Plots For Self-Report Data**


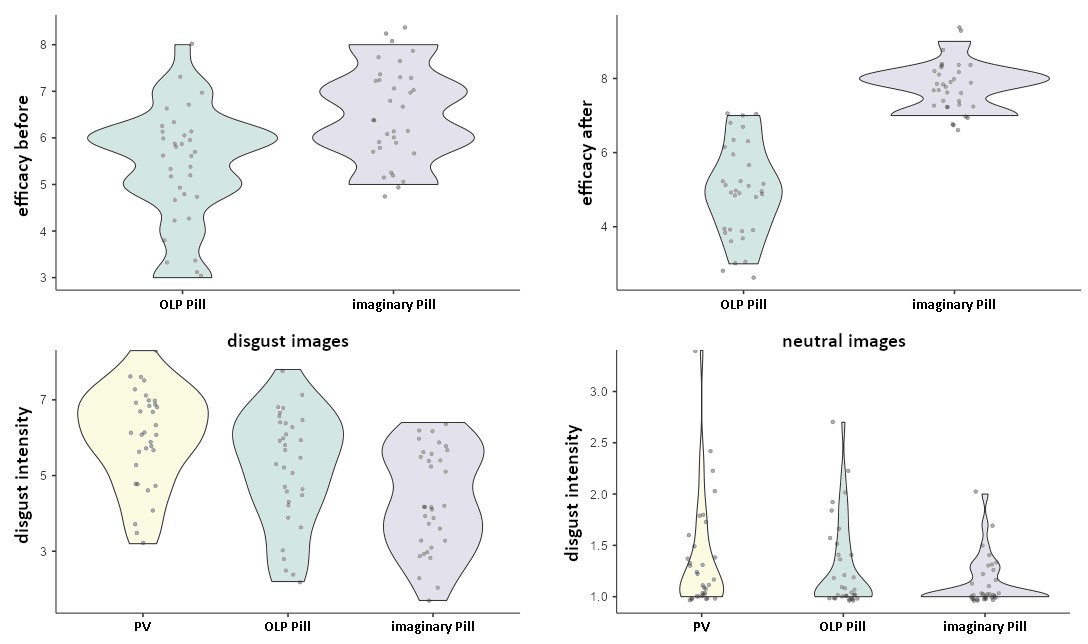


**Supplementary Figure S3: Bar Plots** C**omparing OLP Pill, Imaginary Pill, And Passive Viewing For Pallidum And Insula**


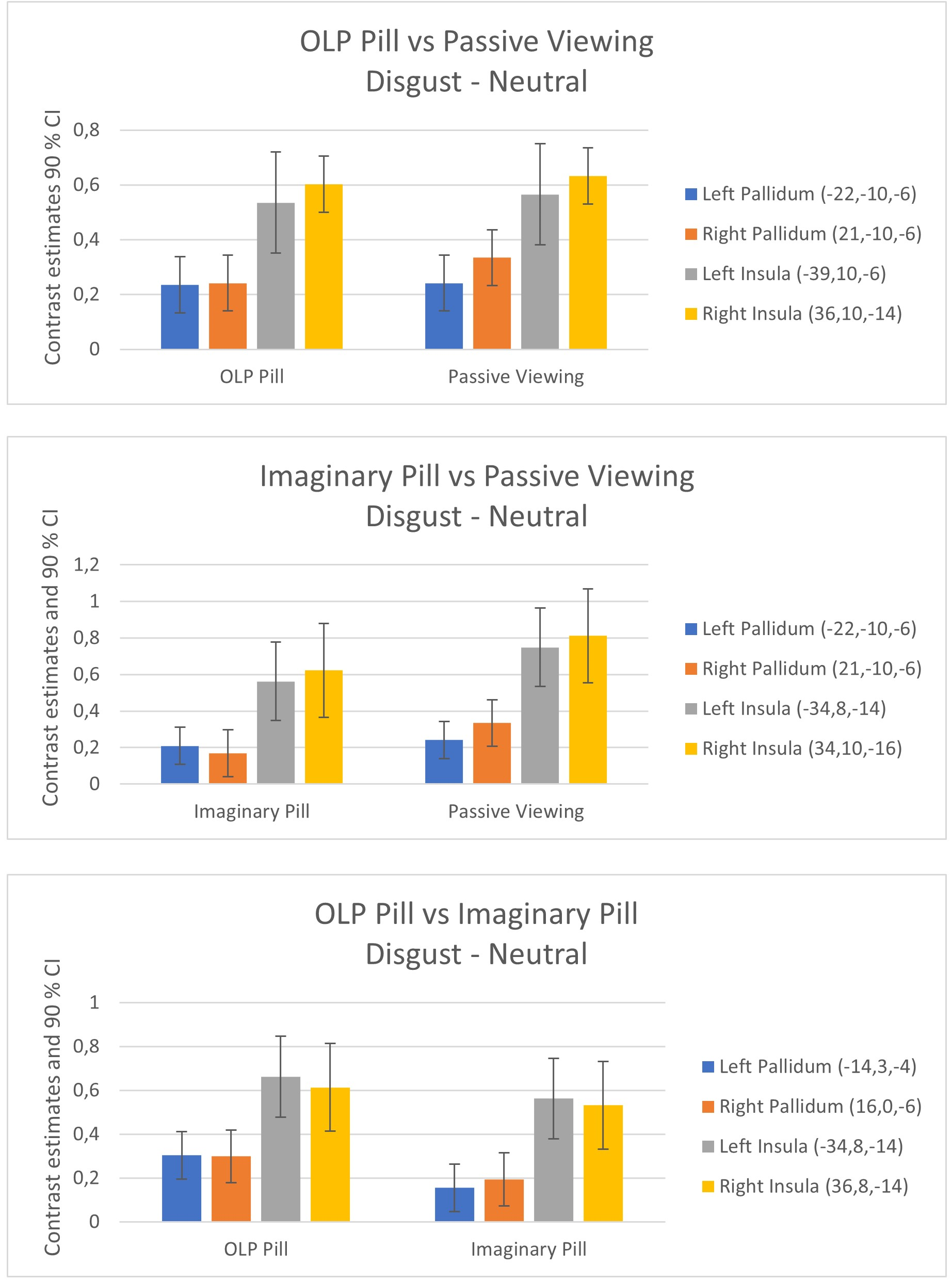

Supplement: nsae021_Supp [file nsae021_supp.zip › scan-23-263-File006.docx]
